# Machine Learning-Driven Metabolomic Biomarker Discovery in Glioblastoma: Advances, Challenges, and Future Directions

**DOI:** 10.3390/ijms27093842

**Published:** 2026-04-26

**Authors:** Tiffany Shih, Rawad Hodeify, Jasprit Kaur, Mohammad Alnuaimi, Orwa Aboud

**Affiliations:** 1 Department of Neurology, University of California, Sacramento, CA 95817, USA; 2 Department of Biotechnology, School of Arts and Sciences, American University of Ras Al Khaimah, Ras Al Khaimah 72603, United Arab Emirates; 3 University of California, Davis, CA 95616, USA; 4 UC Davis Comprehensive Cancer Center, University of California, Davis, Sacramento, CA 95817, USA; 5 Departments of Neurological Surgery, University of California, Davis, Sacramento, CA 95817, USA

**Keywords:** machine learning, glioblastoma, metabolomics, biogenic amines

## Abstract

Glioblastoma (GBM) is an aggressive tumor type known to recur after maximal safe surgical resection followed by concurrent radiation therapy (RT) and chemotherapy (temozolomide—TMZ), and adjuvant TMZ maintenance chemotherapy. It exhibits high intratumor heterogeneity within a single specimen, and thus clinical management remains a challenge due to its rapid progression and high recurrence rate. Machine learning algorithms are currently being implemented in biomarker discovery to develop accurate predictive models that can guide clinical decision making. Emerging evidence identifies metabolomics as a critical player in understanding tumor metabolism and progression. Machine learning computation models have been instrumental in GBM classification and biomarker discovery, as well as the evaluation of tumor staging. Metabolomic profiling of biogenic amines in the setting of surgery, chemoradiation, and understanding relapse also suggests a coordination between metabolic pathways and tumor stage. Many challenges in machine learning and metabolomics-based approaches for disease classification remain due to the dimensionality of datasets, as well as identifying more streamlined panels of metabolite biomarkers. The purpose of this review is to showcase the recent developments in the applications of machine learning in metabolomics as a promising approach to enhancing the biomarker discovery process for future classification and interpretation of patient response to therapies for GBM management in the clinical setting. It also presents the major challenges of implementing machine learning approaches in GBM management and its future directions.

## 1. Introduction

Managing glioblastoma remains a clinical challenge due to its rapid progression and high recurrence rate. Recent evidence suggests that metabolomics offers critical insight into the metabolic reprogramming that underlies tumor progression. Notably, metabolic profiling of patients before the first surgery and relapse surgery, as well as before and after chemoradiation, has revealed significant shifts in metabolite levels, indicating their unique processes behind pathways involved in tumor growth and progression. When combined with machine learning (ML) models, these metabolic findings can be utilized to predict tumor progression, stratify patient risk, and personalize treatment strategies. This review provides a comprehensive and current overview of the machine learning-driven metabolomic approaches applied to GBM research. Unlike prior reviews that discuss glioblastoma metabolomics or machine learning in broader terms, this review specifically focuses on the intersection of these two fields. We emphasize three distinguishing features: (i) integration of metabolomic changes across clinically relevant treatment stages, including pre-surgical, post-surgical, and post-chemoradiation settings; (ii) critical evaluation of how machine learning methods interact with metabolomic data structure, feature selection, and validation strategies; and (iii) focused discussion of the translational barriers that currently limit clinical implementation, including cohort size, preprocessing variability, external validation, and interpretability. By framing the field through these linked biological, computational, and translational dimensions, this review aims to provide a more clinically actionable perspective on ML-driven metabolomic biomarker discovery in glioblastoma. Additionally, in this review, we aim to synthesize the challenges reported in different studies for ML-based metabolomics across GBM and different diseases. Finally, we propose future directions to improve the integration of metabolomics and machine learning in this field of research.

## 2. Metabolomics as a Window in Glioblastoma Biology

Metabolomics plays a critical role in understanding tumor metabolism by providing comprehensive profiles of the most direct metabolic alterations occurring in tumor cells. This process highlights the identification of specific metabolic pathways that are affecting tumor progression. Additionally, intra-tumor heterogeneity has been shown to contribute to the tumor’s aggressive behavior and poor prognosis. The analysis of metabolite profiles in patients with glioblastoma has identified alterations in key metabolic pathways such as the TCA cycle, tryptophan metabolism, the pentose phosphate pathway, and serine and lipid biosynthesis [1]. Several of the altered metabolites belong to the tryptophan metabolic pathway. Previous reports demonstrated that glioblastoma cells obtain their source of energy by utilizing the TCA cycle and aerobic glycolysis [2]. Furthermore, the synthesis of serine and lipids, plus the pentose phosphate cycle, provides cofactors such as NAD to glioblastoma cells, which yields resistance to radiation [2]. Recent findings have also elucidated that nicotinamide phosphoribosyltransferase (NAMPT) can be transferred via microvesicles from radio-resistant glioma stem cells to other tumor cells, leading to elevated intracellular NAD^+^ in the recipient cells [3]. Moreover, increased NAD^+^ levels have been shown to further upregulate NAMPT expression, leading to a positive feedback loop that reinforces the resistance of cancer cells to radiation.

## 3. Machine Learning in Biomarker Discovery and Use of ML in Glioblastoma Research

### 3.1. Machine Learning-Based Survival and Prognosis Prediction

**Figure 1:**
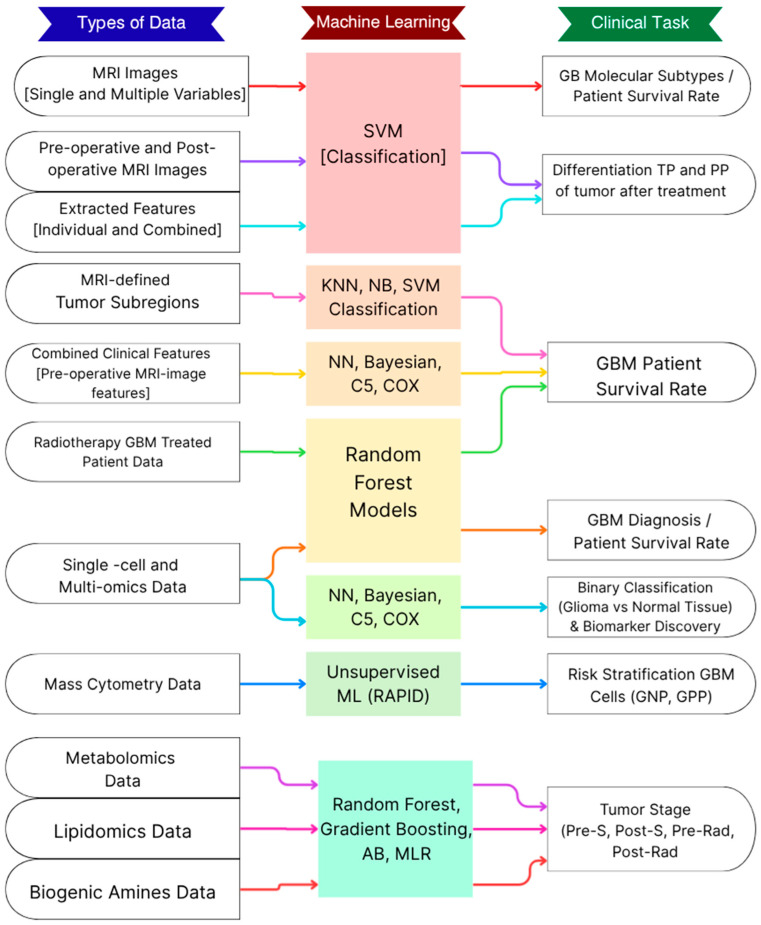
**Machine Learning in Glioblastoma Research.** The figure presents an overview of the types of data used for machine learning algorithms to complete different clinical tasks. These tasks include predicting the patient’s survival rate, classification of glioma against normal tissue, prediction of the tumor stage as compared to pre/post-surgery and pre/post-radiation, and classification of glioblastoma molecular subtypes. Classification algorithms used for the clinical tasks include SVM (Support Vector Machine), KNN (K-Nearest Neighbors), NB (Naïve Bayes), Decision Trees (C5), Survival Models (Cox), Multinomial Logistic Regression, and Random Forest. In addition, an unsupervised machine learning algorithm was used for risk stratification of glioblastoma cells using mass cytometry data, with the RAPID framework applied for an overfitting check.

Implementing machine learning algorithms on imaging data has been the most common approach used in disease management in GBM patients. These methods have been improved by leveraging new technologies in MRI imaging, as well as improvements in the algorithms themselves. However, several downsides remain a matter of continuous discussion, and this impedes its translation into clinical practice. In this section, we assess studies implementing ML in glioblastoma management, presenting significant benefits while highlighting several limitations. In addition, we included important criteria to be considered, such as types of data, feature selection, and validation approaches (Figure 1). The search for literature was conducted in PubMed to identify articles that discuss glioblastoma, machine learning, and biomarkers. Because this article is a narrative review rather than a systematic review, studies were identified through targeted searches in PubMed using combinations of the terms “glioblastoma,” “machine learning,” “metabolomics,” “biomarker discovery,” and “disease classification.” Priority was given to studies directly relevant to glioblastoma that applied machine learning to metabolomic, imaging, proteomic, transcriptomic, or multi-omics datasets in the context of classification, prognosis, treatment response, or tumor stage evaluation. Additional relevant references were identified from the bibliographies of selected articles.

Using MRI imaging data prior to surgery, ML algorithms helped achieve accurate and reproducible prediction of patient survival. In addition, ML tools allowed prediction of glioblastoma molecular subtypes with an overall accuracy of 76% [4]. Rather than a selected set of features, this study included all features in MRI imaging data. For predicting survival rate, Macyszyn et al. used the Support Vector Machines (SVM) algorithm retrospectively on a cohort of 105 patients, followed by a prospective cohort of 29 patients with newly diagnosed glioblastoma [5]. The accuracy of predicting short/medium/long survivors was approximately 77% and 79% in the cross-validated retrospective and prospective cohorts, respectively. The accuracy of the 18-month survival SVM model was 88.57% in the retrospective study and 83.33% in the prospective study. Interestingly, the accuracy of the prediction model was not improved by the addition of tumor molecular subtype estimated by quantitative PCR data to the features. This study demonstrated the benefits of applying machine learning models to imaging data that incorporate multiple variables, rather than relying solely on selected imaging features. On the other hand, the quantitative PCR-based classifier achieved 75.76% accuracy in predicting the four glioblastoma molecular subtypes.

The study by Zhou et al. implemented SVM, KNN, and NB classifiers using data from MRI-defined tumor subregions to predict the survival of GBM patients [6]. The tumor region was segmented based on intensity level using contrast-enhanced T1-weighted and T2-weighted sequences via the automatic threshold selection algorithm. Spatial mapping was done to define tumor subregions from three multiple MRI inputs: T1-CE, T2-w, and FLAIR. For each pair, two groups were created: the Enhanced High (EH) signal group and the Enhanced Low (EL) signal group. Quantitative Group Difference Features (GDFs) were then extracted from these two regions for each pair. The selected features were determined based on the predictive weight for short and long-term survival prediction using a supervised forward feature ranking approach [6]. The training accuracy results for feature selection were determined on each training fold, excluding the leave-one-out set used for evaluation. Once the features for each patient were chosen, the computed features were tested in leave-one-out (LOO) cross-validation using three algorithms for classification, KNN [7], NB [8], and SVM. The KNN algorithm assigns a new data point to the class that holds the majority of data points that fall in the nearest neighbors [7]. The predictive performance of single features on survival was compared with mapped features determined from a pair of habitats tested in Dataset 1, which consisted of 32 patients (a total of 594 tumor slices) with histologically proven GBM collected from the TCGA database. The predictions were validated using Dataset 2 with 22 de-identified patients (261 tumor slices) from an independent institution. The results demonstrated stronger predictive power with spatial correlations of features compared to single feature sets in both datasets for the three algorithms, with accuracy above 80%. However, superior accuracy and sensitivity were reported for SVM with validation in dataset 2.

Jajroudi et al. combined clinical features with pre-operative MRI image features to predict GBM patient survival in a dataset of 55 patients with a mean age of 54.7 [9]. The MRI features were extracted using a fully automated classification tool for brain tumors called Brain Tumor Image Analysis (BraTumIA). The clinical features included Karnofsky Performance Status (KPS), age, gender, chemotherapy, and radiation therapy. Four algorithms for survival prognosis were tested, including NN [10], Bayesian [11], C5 [12], and Cox. Model performance, measured by accuracy, sensitivity, specificity, and area under the curve, demonstrated good performance for NN, C5, and Bayesian, with superior performance for the C5 algorithm. The study concluded that using high-performance models to identify important features can aid in prognosis and treatment management.

A study by Akbari et al. used preoperative and postoperative multiparametric MRI scans to develop an SVM-based classification model to differentiate true progression (TP) from pseudo-progression (PsP) of the tumor after treatment [13]. The quantitative imaging phenomic (QIP) features were extracted from regions of interest defined in the pre-surgery multi-parametric MRI (mpMRI) images that correspond to the resected tissue in post-surgery images. Two types of features were extracted with a deep learning approach, and a priori selected (APS) features extracted using Cancer Imaging Phenomics Toolkit [14]. A supervised classification model relying on the Support Vector Machines (SVM) algorithm was developed using extracted features from individual extraction methods and combined features. With deep learning extracted features, the classification model for PsP vs. non-PsP recorded an accuracy of 87.5% and 78.26 TP vs. non-TP, while the accuracy with APS features was 86.96% and 78.26, respectively [13]. The performance of the classification models was tested first on a training cohort of 40 patients and a separate testing cohort of 23 patients. Performance was also tested on all 63 patients using a leave-one-out cross-validation (LOOCV) method with APS features only. The model achieved 87.30% accuracy for ‘PsP vs. non-PsP’, and 84.13% for TP vs. non-TP. Interestingly, when combining deep learning and APS features, the accuracy for the model decreased for the ‘PsP vs. non-PsP’ classification and was the same for the ‘TP vs. non-TP’. Finally, the model was validated using a training set from a separate institution and tested on a cohort from a third institution. The model successfully diagnosed 7 out of 10 TP patients and 8 out of 10 PsP. The estimated classification of SVM models was also supported by the positive correlation with scores obtained from separate support vector regression (SVR) models and histopathological scores, demonstrating the clinical validity of the workflow. Several limitations can be highlighted for this study. The features extracted are from multi-parametric MRI images, which might not be a routine imaging protocol. However, this allows a comprehensive approach to retrieve many features reflecting anatomical and functional changes. The pre-processing methods and algorithms used in feature extraction are also characterized by considerable variability, which can influence feature differentiation and quantity. The reason for the decreased accuracy with combined features is unclear, and it remains to be determined whether training a deep learning classifier could improve performance.

The adaptation of such an MRI-based prediction framework in clinical settings faces several challenges. First, although the scan is painless, there are barriers for the patient to complete the imaging in situations such as the existence of implanted medical devices that are not MRI-compatible. In addition, several patients may suffer from anxiety due to claustrophobia or physical limitations from prolonged positioning while in the MRI machine, leading to an inability to tolerate acquisition of complete MRI sequences. Secondly, using MRI images as the sole source of data in prediction models may not allow for consideration of factors such as MGMT status, tumor or patient immune system gene expression, and aspects of tumor metabolism such as metabolic and lipidomic profiling and cellular biological pathways. An imaging-based prediction framework without integration of the personalized context of patient disease-specific profile at the genetic, proteomic, and metabolomic levels of the tumor and patient immune physiology limits the interpretability and precision of the developed models.

Peeken et al. implemented ML tools to develop prediction models for overall survival (OS) and progression-free survival (PFS) on retrospectively analyzed data from 189 patients with GBM treated with radiation therapy (RT), among whom 149 patients received concomitant radiochemotherapy [15] following the Stupp et al. protocol [16]. Seven random forest survival models were developed using single and combined clinical, pathological, imaging, and treatment features. The combination of all feature classes of clinical, pathological, imaging, and treatment features achieved the best performance in predicting OS with 96% and 73% accuracy on the training and testing set, respectively. Similarly, the combined featured model with clinical/pathological/imaging features recorded the highest performance in PFS with 79% [95% CI: 0.72–0.85] and 71% [95% CI: 0.60–0.81] accuracy with the training and the testing dataset, respectively. Among the models using individual features, the model with MRI-based features demonstrated the highest accuracy, while the predictive power using clinical and pathological features individually was the lowest. This study demonstrated the prognostic benefit of machine learning prediction models with combined MRI-based features and clinical or pathological features. However, several challenges remain to be addressed, including variability in the imaging equipment and protocols and the diversity of methods for pre-processing data.

### 3.2. Machine Learning Models for GBM Classification and Biomarker Discovery

Several other studies incorporated multi-omics data to develop machine learning algorithms to classify patients with GBM and discover relevant biomarkers. Through analysis of RNA profiles of GBM patient tissues included in TCGA and the CGGA databases, Xu et al. identified 17 differentially regulated genes involved in proneural–mesenchymal transition (PMT) in GBM patients [17]. The expression levels of 17 proneural–mesenchymal transition-related genes (PMTRGs) were used to construct a prognosis model using three algorithms: the Lasso algorithm, multivariate Cox regression, and Lasso modeling followed by Step function calculation. The optimal performance measured by decision curve analysis was demonstrated for the multivariate Cox analysis model with AUC (0.705, CI: 0.632–0.778) at year 1 and AUC 0.773 (CI: 0.683–0.862) at year 2. Furthermore, cluster analysis of the 17 PTMRGs divided 153 patients with GBM into three subclusters, with C1 and C2 clusters having significantly shorter survival time than those in the C3 cluster. Importantly, functional enrichment and pathway analysis demonstrated that shorter survival time is positively correlated to the epithelial–mesenchymal transition of the tumor and to markers of angiogenesis, hypoxia, fatty acid metabolism, inflammatory response, and IL6-JAK-STAT3 signaling. Analysis of the PMTRGs in single-cell sequencing from four GBM patients in the GSE86645 dataset also showed that PMT with a poor prognosis is positively correlated to PMTRGs. Interestingly, the expression of PMTRGs is localized to recruited immune cells and to blood vessels, not in the tumor itself, suggesting a role for these genes in the tumor microenvironment.

Wan et al. used single-cell sequencing data from a tumor core sample and normal-appearing brain tissue surrounding the tumor core, included in the TCGA and GEO databases [18]. Several supervised machine learning models showed excellent performance in binary classification with Partial Least Squares Regression (PLS), NaiveBayes (NB), Gradient Boosting (GB) Machine, Generalized Linear Model, Random Forest, and Support Vector Machine (SVM) showing the least prediction errors and highest predictive performance. This analysis demonstrated a strong correlation of GBM patients’ survival with the expression of MS4A6A, which is one protein within the membrane-spanning 4A gene family. It was initially identified as a biomarker associated with the progression of neurodegenerative diseases [19,20]. The diagnostic value of MS4A6A was examined using TCGA-GBM and TCGA/GTEx-GBM datasets and was shown to be statistically significant with AUCs of 0.995 and 0.987 with 95% CI in the two datasets, respectively. The interpretability of the findings was further supported by the positive correlation of the biological function of MS4A6A revealed with activation of arachidonic acid metabolism and significant inhibition in lysine degradation. In addition, the high MS4A6A expression group was enriched in apoptosis, T cell receptor signaling, and other pathways [18].

Leelatian et al. developed the Risk Assessment Population IDentification (RAPID) algorithm, an unsupervised machine learning algorithm that can identify risk stratifying glioblastoma cells using mass cytometry data from 28 patients with IDH-wild type GBM [21]. Single cell suspensions were collected from resected tissues and stained with a panel of antibodies to detect the expression of known cell surface proteins, intracellular proteins, and phospho-signaling events. The algorithm identified 43 phenotypically distinct cell clusters and then examined the status of each patient’s high risk or low risk for a particular cluster. The RAPID algorithm identified four Glioblastoma Negative Prognostic (GNP) and five Glioblastoma Positive Prognostic (GPP) clusters that were associated with overall survival. The RAPID algorithm was also able to show good performance in predicting positive and negative prognostic populations of cells associated with relapse in a different dataset, including B-cell leukemia cells. The RAPID algorithm uncovered genes associated with negative GBM prognostic cells and decreased overall survival, including neural-lineage proteins S100B and SOX2, proteins involved in cell survival and proliferation, such as p-STAT3 and p-STAT5, and genes with positive prognostic cells and longer overall survival, such as EGFR and CD44 proteins. Interestingly, the RAPID algorithm was able to identify prognostic signatures in a separate flow cytometry dataset in a different disease setting. The advantages of the method by Leelatian et al. are in identifying prognostic clusters in single cells from GBM patients and determining their correlation with patient survival. However, several challenges exist. First, the flow cytometric data includes a library of proteins that are known to be involved in tumor biology. However, it remains highly likely that other potential markers are key determinants of tumor progression that can yield additional subsets of cells within the tumor. Secondly, there is batch variability of the different antibodies for labeling. Thirdly, stratification of tumor subpopulations and correlation with the hazard ratio of death limits the possibility of using this in treatment outcome and response.

Kumari and Kumar used machine learning and computational approaches to identify several biomarkers from non-cellular secretory machinery and post-translational modifications involved in GBM pathogenesis [22]. Using data from TCGA and GEPIA2.0 (Gene Expression Profiling Interactive Analysis) and OSgbm (An Online Consensus Survival Analysis Web Server for Glioblastoma) web tools, the study demonstrated a strong correlation of overexpression of bone morphogenetic protein 1 (BMP1), cathepsin B (CTSB), lysyl oxidase (LOX), procollagenlysine, and 2-oxoglutarate 5-dioxygenase 1 (PLOD1) with poor overall survival. Additionally, the study identified biomarkers connected with response to treatments in responders versus non-responders of GBM patients. Improvement following TMZ therapy was associated with enhanced expression of CTSB (AUC = 0.648, *p* = 0.008) and Von Hippel–Lindau VHL (AUC = 0.667, *p* = 0.00130). Responders to TMZ and chemotherapy demonstrated decreased expression of Ubiquitin Conjugating Enzyme E2 H (Ube2H) (AUC = 0.635, *p* = 0.012) and Histone acetyltransferase 1 (HAT1) (AUC = 0.576, *p* = 0.0092). Furthermore, the study predicted lysine acetylation sites and associated HAT enzymes using deep learning methods. The study presents potential prognostic markers for treatment and survival, but is limited due to the marker validation required to map to relevant biomarkers for therapeutic strategies.

Fontanilles et al. investigated glioblastoma progression through a longitudinal multi-omics liquid biopsy study [23]. In this case–control study, 67 subjects were selected from the GLIOPLAK trial (ClinicalTrials.gov Identifier: NCT02617745). The subjects comprised 34 glioblastoma patients and 33 healthy controls. The blood samples for each patient were collected at several time points: before the treatment (which is the baseline), at week 6 after concomitant radio chemotherapy, and at tumor progression. This study targeted 188 metabolites and 183 proteins. At each sampling point, differential expression analysis of 140 metabolites and 131 protein levels was conducted between patients and controls. For the predictive machine learning, the dataset was separated into 80% training and 20% testing sets. A random forest classification model was used with AUC and ROC curves as performance metrics. One of the key findings of the study is that five metabolites (Pimeloylcarnitine, Leucine, Asparagine, LysoPC a C18:0, PC ae C40:3) and five proteins (NPY, KLK13, SCLY, S100A4, CXCL17) had an AUC above 0.74 with 95% CI. These ten combined features accurately classified patients and controls in the test set with an AUC = 1.0 (95% CI). Notably, NPY and Pimeloylcarnitine demonstrated a good performance individually with an AUC close to 0.95 (95% CI). The design of this longitudinal study allowed the observation of the variation of omics features over time, after treatment, and at recurrence. Even though the cohort of this study is relatively small, with only 34 patients, it demonstrates potential metabolite biomarkers as a promising non-invasive approach for GBM diagnosis and predicting survival outcomes.

A comparative summary (Supplementary Table S1) of the reviewed studies argues against monotypic ML in GBM. Instead, task-specific built models are recommended for GBM diagnosis and prognosis. For the prediction of patient overall survival, several models such as SVM, C5, NB, and Cox demonstrated good performance, with the majority including MRI data. However, models combining MRI data with clinical and pathological data demonstrated superior performance with prognosis, such as true progression versus pseudo progression and low/high risk patients. Additionally, feature selection plays a crucial role in improving the model’s reliability and dimensionality reduction, with sequential or combined feature selection approaches proving to consistently improve the model’s predictive performance. Several studies relied on stratified k-fold cross-validation or leave-one-out cross-validation (LOO-CV) to address the risk of model overfitting. However, many of the studies were missing external validation, while a few utilized existing available databases. This remains a crucial step to ensure that the results are robust and reproducible across different cohorts. Finally, models using transcriptomics data from tumor and normal-appearing brain tissue demonstrated good performance in binary classification [24]. On the other hand, models with proteomics and metabolomics data showed superiority in context-dependent prediction of GBM, such as in the GBM predictions per gender [25,26].

## 4. Metabolomic Profiling and Machine Learning in GBM Patients

Untargeted and targeted metabolomics have been promising approaches used for the discovery of early prediction, diagnostic, and prognostic markers [27]. The comprehensive coverage of untargeted metabolomics and modern high-throughput workflows makes ML tools very useful in discovering hidden patterns and presenting accurate predictions in a timely manner. Although AI-assisted metabolomics studies started with a few publications in the early 2000s, they climbed to hundreds of publications in a few years. Tasci et al. studied the metabolomic dataset from 107 GBM patients between 2005 and 2023 [25]. Metabolomic data obtained from serum samples were collected prior to chemoradiation therapy (CRT) and after its completion. The authors employed individual and hybrid feature approaches using Least Absolute Shrinkage and Selection Operator (LASSO), Minimum Redundancy Maximum Relevance (mRMR), and rank-based selection approaches [24] to categorize the pre–post-CRT, 12-month, and 20-month overall survival (OS) statuses. The study compared six different ML algorithms (SVM, LR, KNN, RF, AdaBoost, and Vote), with and without feature selection using five-fold stratified cross-validation. With single feature selection, the LR model with LASSO feature demonstrated the best performance, with an 89.192% accuracy in classifying the pre–post-CRT status. Lower accuracy was detected when feature selection was not implemented with six models. For classifying the 12-month OS status, the voting ensemble model without using the FS method gives the best result, with a 79.933% accuracy value for the 12-month OS status. However, the best accuracy (64.474% accuracy) for the 20-month OS classification was obtained with an SVM model and LASSO feature selection. Using the hybrid feature selection approach, the best performance (96.711% accuracy) for the pre–post-CRT metabolomic data was obtained with a voting ensemble model with 48 features and a minimum weight of 6. Similar performance (96.24%) was detected with the LR model with the same features and weight. For classification of samples in the 12-month OS-based dataset, the LR model with hybrid feature selection achieved the highest performance (92.093%) with 46 features and a minimum weight of 4. In the 20-month overall survival classification, the highest accuracy (86.910%) with hybrid features was achieved with the voting model using 33 features and a minimum weight of 7. The study identified 48 compounds associated with exposure to concurrent chemoradiation. Although 35 compounds were identified with a confidence level of 3, the two top-ranked compounds alone contributed to 86% accuracy. The first compound had no biological annotation, and the second one was biologically annotated as Inulicin, but subsequent analysis identified it as Iclaprim, or 3-(4-(2-Dimethylamino-1-methylethoxy)phenyl)-1H-pyrazolo(3,4-b)pyridine-1-acetic acid. In the 12-month survival dataset, 2-Furoate and Thymine could be distinguished between patients surviving by 12 months after diagnosis with an accuracy of 80% and a confidence level of 3. The analysis also identified 5-hydroxymethyluracil to be differentially altered between GBM and healthy individuals in plasma [28]. Additional compounds highly matching with authentic standards were identified in CRT (alanine/sarcosine guanine, chenodeoxycholyglycine), 12-month OS (glycine derivative, 2-hydroxyhippuric acid, pantothenic acid or vitamin B5, and caffeine), and L-threonine+Na was identified for 20-month OS.

In a more recent study, Tasci et al. implemented ML models with combined feature selection and weighting methodology (GLIO-Select) on serum proteomics and metabolomics data to identify proteins and metabolites associated with male and female labels in high-dimensional proteomic, metabolomic, and molecular data sets [26]. Using rank-based weighting schemes (1 and 2 to LASSO and to mRMR) on samples from the local serum-based preCRT proteomic dataset, SVM also obtained a 100% accuracy rate with the least number of features. LR also obtained a 100% accuracy rate, but with a larger number of selected features. K-NN, RF, and voting-based ensemble learning models achieved slightly lower performance compared to LR and SVM. The testing accuracy of models in the CPTAC tissue-based proteomic dataset demonstrated that the best performance is for SVM and KNN with 5 selected proteomic features. Testing the GLIO-Select methodology on the preCRT-based metabolomic dataset showed the highest performance for the LR algorithm (91.6% accuracy), while the best result obtained with the CPTAC metabolomic dataset was for LR and voting-based ensemble models with 80% accuracy. With the TCGA-GBM dataset from tumor tissues, the best accuracy (63.6%) for male/female label classification with the GLIO-Select approach was achieved using SVM and RF models. Using the selected feature names, the previously tested algorithms employed for the female/male dataset-based classification, with and without feature selection using five-fold stratified cross-validation, demonstrated that using combined feature selection (LASSO and mRMR) achieved better results across the five datasets. The best result for classifying male/female samples was given with the local proteomic preCRT-based dataset, followed by the Local Level One PreCRT-based metabolomic dataset. The most significant proteomic features (96.3% ACC) characterizing males and females in the local proteomic dataset were Benign Prostate-specific Antigen (BPSA), pregnancy zone protein (PZP), Human Chorionic Gonadotropin (HCG), and Follicle-stimulating hormone (FSH), while RPS4Y1 and DDX3Y were the most discriminative two proteomic features (97% ACC) for the CPTAC dataset. Glutamine, threonine, and N-formylglycine were the most important features (79.5%) in the local serum metabolomic dataset for male and female classification. The three most discriminative and significant features (63.6% ACC) in the TCGA-GBM dataset were EGFR, FAT4, and BCOR. The results of this study underscore the superiority of proteomics and metabolomics in context-dependent prediction of GBM, such as in the gender reported in this study.

Xu et al. analyzed non-targeted metabolomics data from brain tissues to study molecular classification of the GBM and survival prediction [29]. The cohort included 10 control male and female non-tumor patients, four males and six females with IDH mutation, and eight males and two females with IDH-wild-type GBM. Sample visualization based on the three components identified three metabolites: itaconic acid, dulcitol, and 5-aminolevulinic acid, with the highest contribution in subclassifying control, WT, and IDH-groups. Six ML models (RF, NN, C4.0, SVM, DT, CIT) were then established using the three biomarkers identified and were evaluated using confusion matrices. The RF model achieved the highest accuracy of 0.787 (95% CI: 0.780–0.795), followed by the Neural Network (0.723, 95% CI: 0.715–0.732), C5.0 (0.723, 95% CI: 0.714–0.731), and SVM (0.717, 95% CI: 0.708–0.725). Lower accuracy was detected with Decision Tree (0.615, 95% CI: 0.606–0.625) and Conditional Inference Tree (0.603, 95% CI: 0.594–0.613). Using XGBoost and iterative modeling revealed the top 11 metabolites having a C-index above 90% (95% CI: 0.934–0.991). The 11 biomarkers were cholic acid, citrulline, L-tyrosine, nicotinamide-adenine dinucleotide, uric acid, xylose, creatine, L-histidine, hydrocortisone, uridine-5′-diphosphoglucuronic acid, and butanoic acid. The significance tests of survival curves revealed that all 11 biomarkers, except for butanoic acid, hydrocortisone, and creatine, had a significant impact on the survival of patients with GBM.

Hodeify et al. demonstrated the use of metabolic profiling to compare changes in metabolites in patients before and after surgery, as well as before and after radiation therapy. A total of 157 metabolites were identified through retention index alignment and mass spectral matching [30]. Thus, to analyze alterations in metabolic profiles across different treatment stages, a fold change cutoff of 3.0 in MS intensity was applied, enabling the identification of significantly altered metabolites. This study revealed that post-surgery metabolites, sorbitol and mannitol, decrease, whereas urea, oxoproline, glucose, and alanine significantly increase [31]. The decrease in sorbitol post-surgery is consistent with the fact that post-tumor resections may be associated with decreased oxidation of sorbitol to fructose and decreased production of NAD due to the removal of the tumor. Moreover, high levels of urea, oxoproline, glucose, and alanine after surgery suggest increased protein catabolism and gluconeogenesis, indicating the body is adapting to surgical stress and tumor burden [31].

While metabolic changes were observed in both surgical and chemoradiation treatment phases, patients pre- and post-chemoradiation exhibited a different profile of altered metabolites compared to those evaluated pre- and post-surgery. Metabolomic profiling identified a reduction in 11 metabolites post-radiation [31]. Among these, 6-deoxyglucitol exhibited a significant decrease. Given that 6-deoxyglucitol can be converted to erythritol, it is important to note that erythritol levels were also significantly reduced. This observed decrease is consistent with previous findings indicating the role erythritol plays in brain cancer development, potentially through its involvement in the regulation of hydrogen peroxide. Conversely, the data revealed a post-radiation increase in two metabolites: 2,4-difluorotoluene and 9-myristoleate. Although the involvement of 2,4-diflurotoluene in tumor progression is unclear, the increase in 9-myristoleate can be linked to the alteration of fatty acid metabolism of glioblastoma [31]. Lastly, an important observation from metabolic profiling was the identification of positive correlations between several metabolite pairs, including sorbitol and mannitol, indoxyl sulfate and threonic acid, and gluconic acid and gluconic acid lactone. These correlations suggest that there is coordination of multiple intratumoral metabolic pathways.

### Machine Learning Models for Evaluating Tumor Stage

Machine learning algorithms are valuable tools for evaluating different tumor stages. ML models can be utilized to predict and manage the progression of glioblastoma with high accuracy (Figure 1). Specifically, three classification algorithms—multinomial logistic regression (MLR), gradient boosting (GB classifier), and random forest—have been employed to analyze and classify tumor progression patterns [31]. Random Forest is a method used for classification consisting of various decision trees, but with reduced risk of overfitting. Gradient boosting (GB) is also an effective classification method that can produce accurate predictions by growing decision trees based on the prediction errors of previous trees to improve performance accuracy. Finally, multinomial logistic regression is used to predict an outcome in multiple categories from different dependent predictors.

A performance comparison among the three classification models elucidated that the gradient boosting algorithm outperformed both multinomial logistic regression and random forest. Evaluation metrics, which included accuracy, precision, recall, F1-score, ROC-AUC curve, and confusion matrix, were used to assess and compare the predictive performance of each model [31]. In addition, log-loss was used to evaluate the prediction probability of both the logistic regression and gradient boosting models. This metric quantifies the accuracy of predicted probabilities by measuring the divergence between predicted and actual outcomes. The results of this study indicated that the gradient-boosting model exhibited a lower log-loss compared to that of logistic regression, indicating that its performance was better at predicting tumor conditions in patients with glioblastoma who have undergone repeat surgeries. Another feature that was used to determine the validity and accuracy of each model was a 4 × 4 confusion matrix to classify samples in the correct tumor stage. Out of the three ML algorithms evaluated for classification (multinomial logistic regression vs. gradient-boosting classifier vs. random forest), the gradient-boosting classifier classified 11/12 samples correctly, with the random forest algorithm exhibiting the lowest accuracy [31]. Moreover, all performance scores indicated that gradient boosting outperformed the other two models. These results demonstrated a strong potential for ML models to integrate metabolic signatures when classifying different treatment stages of glioblastoma [31]. This can provide valuable insight into patient prognosis by predicting early recurrence, thereby assisting in the development of individualized treatment plans.

Taken together, these studies suggest that the value of machine learning in glioblastoma metabolomics depends less on the selection of any single algorithm and more on the quality, biological relevance, and reproducibility of the underlying metabolomic features. Metabolomics offers dynamic and clinically meaningful information across treatment stages, but it also introduces challenges related to dimensionality, feature instability, cohort size, and preprocessing variability. As a result, model performance should be interpreted in the context of biological plausibility, robustness across datasets, and external validation, rather than accuracy alone. This interaction between metabolomic complexity and machine learning design represents a central determinant of whether candidate biomarkers can move toward clinical translation.

## 5. Biogenic Amines as Predictive Biomarkers

Recent studies explore the role of biogenic amines in gliomas, including in glioma development, diagnostics, and potential future treatment applications. Biogenic amines are organic compounds found in food, plants, and animals, and are produced through the chemical decarboxylation of amino acids. The role of biogenic amines in gliomas has not been fully explored. Typically, the blood–brain barrier acts as both a physical and enzymatic barrier to prevent circulating biogenic amines from affecting the brain. There are five established biogenic amine neurotransmitters, including dopamine, norepinephrine, epinephrine, histamine, and serotonin. Biogenic amine neurotransmitters are not limited to neurotransmitters but also include essential amino acids and other extracellular peptides. Transport of biogenic amines is regulated by uptake and carriers. Interestingly, biogenic amines have also been described to have direct neuroimmune modulatory effects [32], and some biogenic amines are known for their toxic and carcinogenic properties.

Biogenic amines are involved in diverse physiological pathways, and the relationship between the known roles of biogenic amines in glioma development, diagnostics, and potential future treatment applications remains unclear [33]. Recent studies by Aboud et al. on a small cohort showed that amino acids and unsaturated phosphatidylcholines were significantly upregulated in plasma at glioblastoma recurrence compared with initial diagnosis, whereas unsaturated fatty acids and phosphatidylethanolamines were downregulated [34,35,36]. Inter-specimen differences between metabolomic profiles in GBM tissue and plasma were noted, but no intra-specimen differences at diagnosis and at recurrence, suggesting that longitudinal analysis of metabolomic profiles of GBM compared to patient blood specimens can potentially identify targets for adjuvant treatment based on enrichment of specific metabolites in recurrent GBM tissue [34,36]. Additional studies with larger cohorts are needed to corroborate the significant alteration of metabolites, lipids, and biogenic amines between diagnostic and recurrent states in both tumor and plasma specimens. Various biogenic amines, such as peptides, play different roles in cancer cell growth, glioma cell migration, and invasion. These findings suggest novel targets for the treatment of gliomas in the future. While there have been multiple studies characterizing the metabolomes of various sample types (i.e., GBM tissue, CSF, and serum) and describing candidate metabolite biomarkers, the focus of this review will be on the role of machine learning models as an approach to data classification.

## 6. Challenges in ML and Metabolomics-Based Approaches for Disease Classification

### 6.1. Common Challenges Across Several Diseases

**Figure 2:**
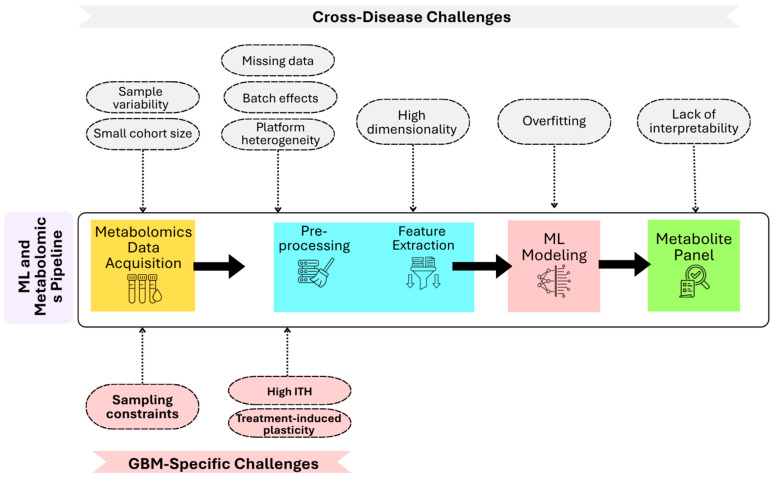
**Challenges in ML and Metabolomics-Based Approaches.** Overview of the ML-metabolomics pipeline and associated challenges. The central pipeline depicts key stages of the ML and metabolomic workflow, including data acquisition and pre-processing, feature selection, ML modeling, and metabolite panel. Cross-disease limitations are shown above each pipeline stage, while GBM-specific challenges are shown below each pipeline stage.

While the integration of ML with metabolomics has shown great promise in enhancing the biomarker discovery process for disease classification, several challenges remain (Figure 2). One of the central challenges of training ML models with metabolomics data is the curse of dimensionality. Like other omics, metabolomics datasets typically have high-dimensional feature spaces, which presents a computational challenge, particularly when studies rely on limited cohort sizes. Several studies in this review highlight this challenge. For instance, Akbari et al. employed training and testing cohorts of 40 patients and 23 patients, respectively [13]. Other studies, such as Jajroudi et al. and Leelatian et al., also relied on small patient cohorts [9,21]. In such settings, where the number of measured metabolites significantly exceeds the number of samples, ML models become prone to overfitting and capturing noise rather than true biological patterns [37,38,39,40,41]. This concern is illustrated by the performance discrepancies reported in Peeken et al., which achieved an overall survival prediction accuracy of 96% in the training set, but decreased to 73% in the testing set [15]. This limitation is often addressed using dimensionality reduction methods. However, that can result in excluding important metabolites from the analysis, which can reduce the ability to detect non-linear effects in metabolomics data [42]. Another major barrier is the issue of data quality, including batch effects, sample variability, and platform heterogeneity. This issue, while commonplace in ML integration with biomedicine in general, is especially prevalent with metabolomics data due to the latter’s high rates of missing data. This is illustrated by cancer metabolomics studies, which note that ML models can suffer from batch effects due to variations in the choice of instrumentation, sample biological source and preparation, and analytical platforms, ultimately masking true biological signals [40,43]. Furthermore, Akbari et al. reported differences in preprocessing pipelines and feature selection strategies, noting that combining deep-learning-derived features with manually selected features did not necessarily improve model performance in a clinically meaningful way [13]. In addition, Peeken et al. highlighted that variability in imaging equipment and preprocessing protocols could introduce bias, while Leelatian et al. emphasized that technical factors such as antibody batch variability and limited marker panels can influence downstream analyses [15,21]. Since this challenge is specific to the pre-processing stage, inadequate data preparation can have significant, cascading, and unfavorable downstream outcomes on the model’s reliability and generalizability.

Furthermore, the high-dimensionality nature of metabolomics datasets requires applying feature-selection strategies to identify a small panel of metabolites to be included in the classification model. Different feature-selection strategies can hinder this process by potentially excluding relevant metabolites or producing a different panel of metabolites depending on the feature-extraction strategy used. Similar performance can be achieved by different feature sets [13]. This challenge limits the establishment of a stable and robust biomarker panel and highlights the importance of externally validating the results. Additionally, regularization and establishing a standardized pre-processing pipeline for each disease type should be explored [34,37,39,41,44]. Algorithms such as SVM, KNN, RF, and NN have shown an ability to achieve high accuracy and a capability of dealing with large, high-dimensional metabolomics datasets [39]. However, these models lack interpretability, specifically those that function as black boxes, namely RF and NN. This limitation is prevalent in the utilization of ML approaches in biological contexts, presenting a major barrier to clinical adoption. To combat this, interpretability can be enhanced by incorporating Explainable AI (XAI) tools such as SHapley Additive exPlanations (SHAP) and Local Interpretable Model-Agnostic Explanations method (LIME). These methods can be applied to trained models to quantify the contribution of individual metabolites to model predictions, allowing researchers to distinguish meaningful metabolic contributions from model-specific artifacts, which aids in verifying the biological significance of the identified metabolite signatures [40,45,46].

Finally, an integrative approach that combines metabolomics with other omics data allows for a more thorough AI-driven analysis of the biological processes of disease, including cancer-associated dysregulation. However, multi-omics analysis presents several problems that contribute to the lack of its integration in ML models. Challenges mentioned before, including dimensionality, batch effects, and platform heterogeneity, are further intensified and compound the already existing computing difficulties. For example, Xu et al. combined machine learning with multi-omics analysis to identify regulators of pro-neural–mesenchymal transition [29]. Additionally, Wan et al., who integrated single-cell and multi-omics data to investigate MS4A6A-associated pathways, demonstrate the potential of these approaches to capture complex disease biology. However, they also highlight key challenges, including differences in data scale, modality-specific preprocessing requirements, missing data across omics layers, and increased dimensionality [17,18]. Hence, an integrative approach, while immensely promising, calls for framework normalization consisting of advanced tools and platforms and requires high computational capabilities to develop reliable multi-omics models [40,47,48,49].

### 6.2. GBM-Specific Challenges

In addition to the general challenges outlined above, GBM presents unique biological and clinical constraints that further complicate ML-based metabolomic approaches. GBM exhibits high intratumor heterogeneity, where a single specimen from a patient contains multiple metabolomic profiles, highlighting spatial heterogeneity within a tumor. Studies [2,31] found that tissue and plasma GBM samples display unique metabolic phenotypes at diagnosis and recurrence. For example, He et al. identified multiple metabolomic profiles within individual GBM specimens [2]. The diverse and dynamic metabolomic profiles across various stages and subregions in the same patient complicate deriving stable and reproducible metabolic signatures by ML models.

Furthermore, the complexity of GBM’s heterogeneous nature is further reinforced by the findings of Bafiti et al., which indicated that GBM undergoes plasticity in response to treatment, further altering the metabolomic profile [50]. This treatment-induced plasticity is especially intensified in GBM compared to other cancers, as patients are almost universally exposed to aggressive and extensive treatments, including surgery, radiotherapy, chemotherapy, and antiepileptic drugs [51].

Moreover, clinical and perioperative factors can introduce additional confounding effects. Perioperative medications and clinical interventions may influence metabolite levels, further complicating the interpretation of metabolomics data [31]. These confounders are particularly relevant in GBM, where patients often receive complex treatment regimens that can independently alter metabolic pathways.

Sampling constraints are another fundamental challenge unique to GBM metabolomic studies. Acquiring tumor tissue samples requires invasive surgical interventions and cerebrospinal fluid (CSF). While these sources provide a more accurate metabolomic picture because of their proximity to CNS tissue, it also presents ethical concerns due to the invasive nature of the collection approach [52]. Therefore, researchers rely on plasma samples as a more practical approach that better enables repeated longitudinal sampling [34,53]. While plasma samples aid in obtaining a more comprehensive, multi-stage picture of metabolomic profiles, the potential variable of the blood–brain barrier (BBB) may confound the representation of the true metabolomic signatures of the local tumor environment. Studies found that differences in GBM-induced impaired BBB integrity and subsequent permeability alterations can affect the metabolomic profile and weaken the correlation between plasma and CSF metabolite signatures [54,55]. This limitation complicates biomarker discovery and affects the dependability and robustness of the resulting biomarker panels.

## 7. Translational Potential of ML-Based Metabolomic Biomarkers, and Future Directions

Machine learning, in combination with metabolomic profiling, holds promise for improving risk stratification and informing future personalized treatment strategies, but remains early in clinical translation. Thus, by leveraging machine learning models, this approach may eventually support earlier detection and longitudinal monitoring of therapeutic response, pending vigorous validation. Furthermore, this approach provides a foundation for integrating metabolomic data and machine learning into clinical decision support systems. While the conducted studies yielded valuable insights, several challenges remain. There is a need for larger, multi-institutional datasets and more robust longitudinal study designs to expand upon these results. Future research should prioritize the inclusion of larger, multicenter cohorts to enhance the generalizability and statistical power of metabolomic analyses. Additionally, the inclusion of control groups would strengthen the study by providing a critical reference point. Another promising approach could be incorporating multi-institutional datasets and integrating metabolomics with proteomics and genomics to enable the discovery of multimodal biomarkers. Recent studies indicate that integrating metabolomic profiles from disease and control samples with proteomic data allows for the development of more robust biomarker panels [56]. Future work could focus on leveraging AI and machine-learning approaches to predict responses to immunotherapy or targeted therapies. Regardless of their promising approach, AI-based clinical decision support systems pose challenges that need to be addressed to ensure seamless integration of these tools in routine clinical care. The level of patient safety and the legal responsibility for diagnostic errors made by these tools remain a significant barrier for physicians who are proactively utilizing the tools in their clinical practice [57]. In addition, even if regulatory frameworks guide the deployment and development of AI-based decision support systems, there remains a challenge of having algorithms consistent with the fast growth of clinical research, periodic maintenance, and systematic re-evaluation after defined time frames [57]. Another important consideration pointed out by Mohyeldin et al. highlights the importance of considering data bias and interpretability issues when implementing AI-based clinical decision support systems. AI-driven clinical solutions must be explainable with regulatory frameworks and integration strategies that consider the current clinical workflows [58]. Until these challenges are addressed, the tools should remain within a scope where clinicians have the authority to override algorithmic recommendations, aligning with clinical reasoning and improved patient outcomes [58]. Cross-dataset validation and external validation have suggested a potential approach to address this limitation. Interpretability is another limitation highlighted in Tasci et al.’s study [26]. The authors in Tasci et al. utilized the feature selection tools such as LASSO and mRMR, which are known for their interpretability. They also proposed SHAP analysis to support the explainability of their results. Another common limitation in metabolomics studies in GBM is the dependency of the mass spectrometry on the mass-to-charge ratio of the compounds, which may present inaccuracies as more than one compound may share a similar mass-to-charge ratio. The limited biological annotation of metabolome compounds using reference libraries yields several unidentified compounds.

## 8. Conclusions

In conclusion, this review demonstrates that metabolomic profiling combined with machine learning can reveal the metabolic alterations associated with recurrent IDH-wildtype glioblastoma. These approaches aid in clarifying the metabolic pathways that contribute to tumor behavior and progression in patients. Furthermore, machine learning modules show promise as predictive tools for determining the phase or state of tumor development. Overall, the findings presented in this review point toward a future in which these strategies may inform future clinical decision-making frameworks in glioblastoma once validated in larger, standardized, and prospectively studied cohorts.

## Data Availability

No new data were created or analyzed in this study. Data sharing is not applicable to this article.

## Supplementary Materials

The following supporting information can be downloaded at https://www.mdpi.com/article/10.3390/ijms27093842/s1.

## Abbreviations

The following abbreviations are used in this manuscript:

AI	Artificial intelligence
CD	Cluster of differentiation
EGFR	Epidermal Growth Factor Receptor
GB	Gradient boosting
GBM	Glioblastoma
IDH	Isocitrate dehydrogenase
JAK	Janus Kinase
kNN	k-Nearest Neighbor
ML	Machine learning
MRI	Magnetic resonance imaging
NAD	Nicotinamide Adenine Dinucleotide
NB	Naïve bayes
NN	Neural Network
OTSU	Optimized maximum between-class variance thresholding method
PCR	Polymerase Chain Reaction
RF	Random forest
SOX	SRY-related HMG-box
STAT	Signal Transducer and Activator of Transcription
SVM	Support vector machine
TCA	Tricarboxylic acid

## References

[1] Bernhard C., Reita D., Martin S., Entz-Werle N., Dontenwill M. (2023). Glioblastoma Metabolism: Insights and Therapeutic Strategies. Int. J. Mol. Sci..

[2] He W.S., Edney M.K., Paine S.M.L., Griffiths R.L., Scurr D.J., Rahman R., Kim D.H. (2023). Untargeted Metabolomic Characterization of Glioblastoma Intra-Tumor Heterogeneity Using OrbiSIMS. Anal. Chem..

[3] Lucena-Cacace A., Otero-Albiol D., Jiménez-García M.P., Peinado-Serrano J., Carnero A. (2017). NAMPT overexpression induces cancer stemness and defines a novel tumor signature for glioma prognosis. Oncotarget.

[4] Macyszyn L., Akbari H., Pisapia J.M., Da X., Attiah M., Pigrish V., Bi Y., Pal S., Davuluri R.V., Roccograndi L. (2016). Imaging patterns predict patient survival and molecular subtype in glioblastoma via machine learning techniques. Neuro Oncol..

[5] Chang C.C., Lin C.J. (2011). LIBSVM: A Library for Support Vector Machines. ACM Trans. Intell. Syst. Technol..

[6] Zhou M., Hall L.O., Goldgof D.B., Gatenby R.A., Gillies R.J. (2013). A Texture Feature Ranking Model for Predicting Survival Time of Brain Tumor Patients. 2013 IEEE International Conference on Systems, Man, and Cybernetics.

[7] Cover T.M., Hart P.E. (1967). Nearest Neighbor Pattern Classification. IEEE Trans. Inf. Theory.

[8] Ng A.Y., Jordan M.I. (2002). On Discriminative vs. Generative classifiers: A comparison of logistic regression and naive Bayes. Adv. Neural Inf. Process. Syst..

[9] Jajroudi M., Enferadi M., Homayoun A.A., Reiazi R. (2022). MRI-based machine learning for determining quantitative and qualitative characteristics affecting the survival of glioblastoma multiforme. Magn. Reson. Imaging.

[10] Gallego V., Insua D.R. (2022). Current Advances in Neural Networks. Annu. Rev. Stat. Its Appl..

[11] Forsberg J.A., Eberhardt J., Boland P.J., Wedin R., Healey J.H. (2011). Estimating survival in patients with operable skeletal metastases: An application of a bayesian belief network. PLoS ONE.

[12] Mardhatillah M., Aidilof H.A.K., Aidilof A. (2025). Comparative Analysis of the C5.0 Algorithm and Other Machine Learning Models for Early Detection of Multi-Class Heart Disease. J. Appl. Inform. Comput..

[13] Akbari H., Rathore S., Bakas S., Nasrallah M.P., Shukla G., Mamourian E., Rozycki M., Bagley S.J., Rudie J.D., Flanders A.E. (2020). Histopathology-validated machine learning radiographic biomarker for noninvasive discrimination between true progression and pseudo-progression in glioblastoma. Cancer.

[14] Davatzikos C., Rathore S., Bakas S., Pati S., Bergman M., Kalarot R., Sridharan P., Gastounioti A., Jahani N., Cohen E. (2018). Cancer imaging phenomics toolkit: Quantitative imaging analytics for precision diagnostics and predictive modeling of clinical outcome. J. Med. Imaging.

[15] Peeken J.C., Goldberg T., Pyka T., Bernhofer M., Wiestler B., Kessel K.A., Tafti P.D., Nusslin F., Braun A.E., Zimmer C. (2019). Combining multimodal imaging and treatment features improves machine learning-based prognostic assessment in patients with glioblastoma multiforme. Cancer Med..

[16] Stupp R., Hegi M.E., Mason W.P., van den Bent M.J., Taphoorn M.J., Janzer R.C., Ludwin S.K., Allgeier A., Fisher B., Belanger K. (2009). Effects of radiotherapy with concomitant and adjuvant temozolomide versus radiotherapy alone on survival in glioblastoma in a randomised phase III study: 5-year analysis of the EORTC-NCIC trial. Lancet Oncol..

[17] Xu C., Yang J., Xiong H., Cui X.T., Zhang Y.H., Gao M.J., He L., Fang Q.Y., Han C.X., Liu W. (2025). Machine learning and multi-omics analysis reveal key regulators of proneural-mesenchymal transition in glioblastoma. Sci. Rep..

[18] Wan F.C., Li Y.L., Zhu J.M., Yu D.D., Liu H.J., Hu B.H. (2024). Exploring the prognostic value and potential therapeutic strategies of MS4A6A in glioblastoma: A comprehensive analysis of single-cell and multi-omics data. J. Cell. Mol. Med..

[19] Karch C.M., Jeng A.T., Nowotny P., Cady J., Cruchaga C., Goate A.M. (2012). Expression of novel Alzheimer’s disease risk genes in control and Alzheimer’s disease brains. PLoS ONE.

[20] Zhang C., Liu H., Tan Y., Xu Y., Li Y., Tong S., Qiu S., Chen Q., Su Z., Tian D. (2022). MS4A6A is a new prognostic biomarker produced by macrophages in glioma patients. Front. Immunol..

[21] Leelatian N., Sinnaeve J., Mistry A.M., Barone S.M., Brockman A.A., Diggins K.E., Greenplate A.R., Weaver K.D., Thompson R.C., Chambless L.B. (2020). Unsupervised machine learning reveals risk stratifying glioblastoma tumor cells. eLife.

[22] Kumari S., Kumar P. (2023). Identification and characterization of putative biomarkers and therapeutic axis in Glioblastoma multiforme microenvironment. Front. Cell Dev. Biol..

[23] Fontanilles M., Heisbourg J.D., Daban A., Di Fiore F., Pepin L.F., Marguet F., Langlois O., Alexandru C., Tennevet I., Ducatez F. (2024). Metabolic remodeling in glioblastoma: A longitudinal multi-omics study. Acta Neuropathol. Commun..

[24] Wang D.H., Fujita Y., Dono A., Rodriguez Armendariz A.G., Shah M., Putluri N., Pichardo-Rojas P.S., Patel C.B., Zhu J.J., Huse J.T. (2024). The genomic alterations in glioblastoma influence the levels of CSF metabolites. Acta Neuropathol. Commun..

[25] Tasci E., Popa M., Zhuge Y., Chappidi S., Zhang L., Cooley Zgela T., Sproull M., Mackey M., Kates H.R., Garrett T.J. (2024). MetaWise: Combined Feature Selection and Weighting Method to Link the Serum Metabolome to Treatment Response and Survival in Glioblastoma. Int. J. Mol. Sci..

[26] Tasci E., Chappidi S., Zhuge Y., Zhang L., Cooley Zgela T., Sproull M., Mackey M., Camphausen K., Krauze A.V. (2025). GLIO-Select: Machine Learning-Based Feature Selection and Weighting of Tissue and Serum Proteomic and Metabolomic Data Uncovers Sex Differences in Glioblastoma. Int. J. Mol. Sci..

[27] Qiu S., Cai Y., Yao H., Lin C., Xie Y., Tang S., Zhang A. (2023). Small molecule metabolites: Discovery of biomarkers and therapeutic targets. Signal Transduct. Target. Ther..

[28] Ferrasi A.C., Puttini R., Galvani A.F., Hamamoto Filho P.T., Delafiori J., Argente V.D., de Oliveira A.N., Dias-Audibert F.L., Catharino R.R., Silva O.C. (2023). Metabolomics Approach Reveals Important Glioblastoma Plasma Biomarkers for Tumor Biology. Int. J. Mol. Sci..

[29] Xu P., Chen X., Li Q., Dong Z., Zhu J., Su Z., Zhang Q., Fang K. (2025). Nontargeted metabolomics uncovering metabolite signatures in glioblastoma: A preliminary study on candidate biomarker discovery for IDH subtyping and survival prediction. Front. Oncol..

[30] Fiehn O. (2016). Metabolomics by Gas Chromatography-Mass Spectrometry: Combined Targeted and Untargeted Profiling. Curr. Protoc. Mol. Biol..

[31] Hodeify R., Yu N., Balasubramaniam M., Godinez F., Liu Y., Aboud O. (2024). Metabolomic Profiling and Machine Learning Models for Tumor Classification in Patients with Recurrent IDH-Wild-Type Glioblastoma: A Prospective Study. Cancers.

[32] Belousova O., Lopatina A., Kuzmina U., Melnikov M. (2023). The role of biogenic amines in the modulation of monocytes in autoimmune neuroinflammation. Mult. Scler. Relat. Disord..

[33] Abuaisheh A., Aboud O. (2023). Biogenic Amines in Gliomas: A Comprehensive Literature Review. Front. Biosci.-Landmark.

[34] Aboud O., Liu Y., Dahabiyeh L., Abuaisheh A., Li F., Aboubechara J.P., Riess J., Bloch O., Hodeify R., Tagkopoulos I. (2023). Profile Characterization of Biogenic Amines in Glioblastoma Patients Undergoing Standard-of-Care Treatment. Biomedicines.

[35] Liu A., Aboud O., Dahabiyeh L.A., Bloch O., Fiehn O. (2023). A pilot study on metabolomic characterization of human glioblastomas and patient plasma. Res. Sq..

[36] Liu Y.A., Aboud O., Dahabiyeh L.A., Bloch O., Fiehn O. (2024). Metabolomic characterization of human glioblastomas and patient plasma: A pilot study. F1000Research.

[37] Aboud O., Liu Y.A., Fiehn O., Brydges C., Fragoso R., Lee H.S., Riess J., Hodeify R., Bloch O. (2023). Application of Machine Learning to Metabolomic Profile Characterization in Glioblastoma Patients Undergoing Concurrent Chemoradiation. Metabolites.

[38] Anh N.K., Lee A., Phat N.K., Yen N.T.H., Thu N.Q., Tien N.T.N., Kim H.S., Kim T.H., Kim D.H., Kim H.Y. (2024). Combining metabolomics and machine learning to discover biomarkers for early-stage breast cancer diagnosis. PLoS ONE.

[39] Galal A., Talal M., Moustafa A. (2022). Applications of machine learning in metabolomics: Disease modeling and classification. Front. Genet..

[40] Elguoshy A., Zedan H., Saito S. (2025). Machine Learning-Driven Insights in Cancer Metabolomics: From Subtyping to Biomarker Discovery and Prognostic Modeling. Metabolites.

[41] Xie Y., Meng W.Y., Li R.Z., Wang Y.W., Qian X., Chan C., Yu Z.F., Fan X.X., Pan H.D., Xie C. (2021). Early lung cancer diagnostic biomarker discovery by machine learning methods. Transl. Oncol..

[42] Gomari D.P., Schweickart A., Cerchietti L., Paietta E., Fernandez H., Al-Amin H., Suhre K., Krumsiek J. (2022). Variational autoencoders learn transferrable representations of metabolomics data. Commun. Biol..

[43] Wang W., Zhen S., Ping Y., Wang L., Zhang Y. (2024). Metabolomic biomarkers in liquid biopsy: Accurate cancer diagnosis and prognosis monitoring. Front. Oncol..

[44] Melo C., Navarro L.C., de Oliveira D.N., Guerreiro T.M., Lima E.O., Delafiori J., Dabaja M.Z., Ribeiro M.D.S., de Menezes M., Rodrigues R.G.M. (2018). A Machine Learning Application Based in Random Forest for Integrating Mass Spectrometry-Based Metabolomic Data: A Simple Screening Method for Patients With Zika Virus. Front. Bioeng. Biotechnol..

[45] Colak C., Yagin F.H., Yagin B., Alkhateeb A., Al-Rawi M.B.A., Akhloufi M.A., Aghaei M. (2025). Identification of metabolomics-based biomarker discovery in individuals with down syndrome utilizing kernel-tree model-enhanced explainable artificial intelligence methodology. Front. Mol. Biosci..

[46] Guldogan E., Yagin F.H., Ucuzal H., Alzakari S.A., Alhussan A.A., Ardigo L.P. (2025). Interpretable Machine Learning for Serum-Based Metabolomics in Breast Cancer Diagnostics: Insights from Multi-Objective Feature Selection-Driven LightGBM-SHAP Models. Medicina.

[47] Subramanian I., Verma S., Kumar S., Jere A., Anamika K. (2020). Multi-omics Data Integration, Interpretation, and Its Application. Bioinform. Biol. Insights.

[48] Costantini S., Di Gennaro E., Capone F., De Stefano A., Nasti G., Vitagliano C., Setola S.V., Tatangelo F., Delrio P., Izzo F. (2022). Plasma metabolomics, lipidomics and cytokinomics profiling predict disease recurrence in metastatic colorectal cancer patients undergoing liver resection. Front. Oncol..

[49] Cai Z., Poulos R.C., Liu J., Zhong Q. (2022). Machine learning for multi-omics data integration in cancer. iScience.

[50] Bafiti V., Ouzounis S., Siapi E., Grypari I.M., Theofanopoulos A., Panagiotopoulos V., Zolota V., Kardamakis D., Katsila T. (2023). Bioenergetic Profiling in Glioblastoma Multiforme Patients with Different Clinical Outcomes. Metabolites.

[51] Davis M.E. (2016). Glioblastoma: Overview of Disease and Treatment. Clin. J. Oncol. Nurs..

[52] Costantini S., Di Gennaro E., Fanelli G., Bagnara P., Argenziano C., Maccanico C., Paggi M.G., Budillon A., Abbruzzese C. (2025). Glioblastoma metabolomics: Uncovering biomarkers for diagnosis, prognosis and targeted therapy. J. Exp. Clin. Cancer Res..

[53] Zhou J., Ji N., Wang G., Zhang Y., Song H., Yuan Y., Yang C., Jin Y., Zhang Z., Zhang L. (2022). Metabolic detection of malignant brain gliomas through plasma lipidomic analysis and support vector machine-based machine learning. eBioMedicine.

[54] Riviere-Cazaux C., Carlstrom L.P., Rajani K., Munoz-Casabella A., Rahman M., Gharibi-Loron A., Brown D.A., Miller K.J., White J.J., Himes B.T. (2023). Blood-brain barrier disruption defines the extracellular metabolome of live human high-grade gliomas. Commun. Biol..

[55] Rogachev A.D., Alemasov N.A., Ivanisenko V.A., Ivanisenko N.V., Gaisler E.V., Oleshko O.S., Cheresiz S.V., Mishinov S.V., Stupak V.V., Pokrovsky A.G. (2021). Correlation of Metabolic Profiles of Plasma and Cerebrospinal Fluid of High-Grade Glioma Patients. Metabolites.

[56] Srivastava A., Creek D.J. (2019). Discovery and Validation of Clinical Biomarkers of Cancer: A Review Combining Metabolomics and Proteomics. Proteomics.

[57] Chua I.S., Gaziel-Yablowitz M., Korach Z.T., Kehl K.L., Levitan N.A., Arriaga Y.E., Jackson G.P., Bates D.W., Hassett M. (2021). Artificial intelligence in oncology: Path to implementation. Cancer Med..

[58] Mohyeldin M., Mohamed F.O., Molina M., Towfig M.F., Mustafa A.M.G., Elhussein A.H., Alamin F., Khaja M., Jadhav P. (2025). Artificial Intelligence in Hypertrophic Cardiomyopathy: Advances, Challenges, and Future Directions for Personalized Risk Prediction and Management. Cureus.

