# Supplementary material for: Machine Learning-Driven Metabolomic Biomarker Discovery in Glioblastoma: Advances, Challenges, and Future Directions
Source: Int J Mol Sci. 2026 Apr 26;27(9):3842. doi: 10.3390/ijms27093842 (PMC13163234; doi:10.3390/ijms27093842)
Supplement: Supplementary file 1 [file ijms-27-03842-s001.zip › ijms-4234905-supplementary.pdf]

Supplementary Table S1

Study	Task	Data Type	Sample Size	Training/Testing Split	CrossValidation	External Validation	Machine Learning Algorithm	Overfitting Risk	Feature Selection Strategy	Confidence Interval	Performance Metrics
Macyszyn, L et al	Predicting short/medium/long survivors	MRI images	105 (N=29GBM)	90%: 10%	10-fold	Yes - replication via internal cohort	Multi-dimensional pattern classification method (SVM)	No	MRI Imaging and Clinical Features	95%	Classification into short/medium/long survivors77% (retrospective cohort), 79% (prospective cohort)
Zhou et al.	Predicting survival rate of GBM patients	MRI images	N=32	32 training, each feature independently using a leave-one-out cross validation		Not reported	Feature Ranking Model, SVM Classifiet	No	Feature Ranking Model	Not reported	85% accuracy
Jajroudi et al.	Predicting survival rate of GBM patients	MRI and clinical features	N= 55	Split done but ratio not reported	10-fold	Not reported	ANN, C5, Bayesian, and Cox models	No	Wrapper Feature Selection	Not reported	NN (89.09%) C5(94%) Bayesian (84.5%) COX(49.9%)
Akbari et al	Differentiate true progression (non-PsP) from pseudo-progression (PsP) of the tumor after treatment	MRI images	N=63	Training =43, Testing=20	Leave-one-out cross-validation	independent inter-institutional cohort from a different center (n = 20).	Multivariate pattern classification SVM	No	Sequential feature selection from >1,000 radiomic features; pretrained CNN for deep features	Not reported	PsP vs non-PsP ~87–87%; TP vs non-TP ~78–84%; Inter-institutional ~75%
Peeken et al	Predict overall survival (OS) and progression-free survival (PFS)	MRI-based features, clinical and pathological features, treatment features	N=189 patients	Training =132, Testing=57	Not reported	Not reported	Random Forest algorithm	Yes	VIMP function was applied to calculate feature permutation importance	95% confidence interval	The combination of all features (MRI, clinical, pathological, and treatment achieved the best performance in predicting OS with 96% and 73% accuracy on training and testing set, respectively. Similarly, the combined featured model recorded highest performance in progression-free survival with 79% and 71% ac-curacy on training and

											testing dataset, respectively.
Xu. C et al	Prognostic risk model constructed by three machine learning algorithms: Lasso, multivariate Cox analysis, and Step function	RNA profiles of GBM patient tissues included in TCGA and the CGGA databases	671 LOW-GRADE GLIOMA and 153 GBM tissue sample (TCGA and CGGA datasets)	Unsupervised prognostic modeling	Not reported	Conducted on TCGA and CGGA datasets	Prognostic model based on Lasso, Cox, and Step algorithms	No	Lasso regression, multivariate Cox, and Stepwise selection	Not reported	highest performance with Cox AUC 0.705 (survival at 1 year)
Wan. F et al	Single-cell sequencing data from tumor core sample and normal-appearing brain tissue, **binary classification**	Single-cell sequencing	TCGA-GBM and GTEx cohorts, single-cell dataset	50%:50%	Generalized Linear Model (GLM), Elastic net regression (Elastic net), Gradient Boosting Machine (GBM), K Nearest Neighbours (KNN), NaiveBayes, Logistic Regression (Logit), Support Vector Machines (SVM), Random Forests (RF), Stepwise Linear Discriminant Analysis (stepLDA), and Partial Least	Public datasets are used	Multiple machine learning methods using caret package	No	Multiple machine learning algorithms	95% for AUC Values	ROC,AUC,AUCs of 0.995 and 0.987

					Squares Regression						
Leelatian et al	Identification of risk stratifying glioblastoma cells	Sampled cells	28	unsupervised modeling	unsupervise clustering approach (Ten independent t-SNE analyses were performed on equal numbers of randomly sampled cells from each patient)	Statistical and Biological validation using public data		Yes	t-SNE Map	Not reported	Not reported - calculated Fmeasure
Hodeify et al	Tumor stage classification	Blood Samples	39	70%:30%	Not reported	Not reported	Gradient Boosting, Logistic Regression	Yes	Metabolomic Profiling, Gini Importance	Not reported	GB 92%, lr 88%
Kumari and Kumar	Identify several biomarkers from non-cellular secretory machinery and post-translational modifications involved in GBM pathogenesis	data from TCGA and GEPIA2.0 (Gene Expression Profiling Interactive Analysis) and OSgbm (An Online Consensus Survival Analysis Web Server for Glioblastoma)	163 GBM Tissue and 207 Normal Tissue	NA	NA	NA	Expression Analysis and Correlation Study Analysis	NA	differential gene expression, PPI network analysis	95%	AUC 0.66

Tasci et al	individual and hybrid feature approach using Least Absolute Shrinkage and Selection Operator (LASSO) and Min-imum Redundance Maximum Relevance (mRMR) and rank-based selection approaches	Serum Samples from GBMM Patients	107	Not reported	Stratified 5-fold cross-validation	Not reported	Support Vector Machine (SVM), K-Nearest Neighbors (KNN), Logistic Regression (LR), Adaptive Boosting (AdaBoost), Random Forest (RF), and the voting ensemble model	No	Combined feature selection using LASSO and mRMR followed by rank-based weighting (MetaWise) for dimensionality reduction	Not reported	reported for three tasks: 96.711%, 92.093%, and 86.910%
Fontanilles et al	metabolic and proteomic profiles, classification of GBM and controls	Case-study control selected from GLIOPLAK trial - comediaction and patients' characteristics (Metabolomics and proteomics datasets)	67 (34 subjects biopsy-only patients and 33 controls)	80% - 20%	Stratified 5-fold cross-validation	Not reported	Iterative COX modeling	No	Predictive models using omics features / classification modesl using caret R package (Random Forest as classification algorithm)	95%	These ten combined features (5 metabolites and 5 proteins) accurately classified patients and controls, in the test set, with an AUC=1.0 (95% CI). Notably, NPY and Pimeloylcarnitine demonstrated a good performance individually with an AUC close to 0.95 (95% CI).
Tasci et al	superiority of proteomics and metabolomics in context-dependent prediction of GBM such as in the gender reported in this study.	proteomic and metabolomic profiles from serum biospecimens	109 - highly dimensional dataset	Not reported	Stratified 5-fold cross-validation	Performed against (CPTAC + TCGA datasets)	voting-based ensemble learning model consists of five prediction models, including SVM, KNN, LR, RF, and AdaBoost by applying the soft voting rule	No	Hybrid feature selection + weighting (LASSO + mRMR + GLIO-Select)	Not reported	Accuracy used as primary metric for selected features (80%-100%)
